# Assessing outcomes of full-thickness resection in piecemeal polypectomy scar consolidation of colon adenomas containing cancer

**DOI:** 10.1055/a-2637-2047

**Published:** 2025-09-01

**Authors:** Ivana Radosavljevic, Aamir Dam, Anjuli K Luthra, Luis Pena, Saraswathi Cappelle, Jennifer B Permuth, Seth Felder, Julian Sanchez, Amalia Stefanou, Mark Friedman, Shaffer R Mok

**Affiliations:** 1 1371 Department of Internal Medicine, Emory University, Atlanta, United States; 2 551399 Department of Gastrointestinal Oncology, H Lee Moffitt Cancer Center and Research Institute, Tampa, United States; 3 25139 Gastroenterology, Temple University Hospital, Philadelphia, United States

**Keywords:** Endoscopy Lower GI Tract, Colorectal cancer, Polyps / adenomas / ..., CRC screening, Endoscopic resection (polypectomy, ESD, EMRc, ...)

## Abstract

**Background and study aims:**

The current standard of care for patients who are found to have malignancy within a resected colorectal polyp segment is surgical resection. Our study aimed to illustrate the efficacy and safety of using endoscopic full thickness resection (EFTR) to achieve histologically complete (R0) resection and formal staging in malignant polypectomy scars.

**Patients and methods:**

This was a prospective case series of 14 patients who underwent scar consolidation via EFTR following piecemeal polypectomy or endoscopic mucosal resection (EMR) of a malignant colorectal polyp. Variables collected assessed R0 resection, technical success of the procedure, residual disease within the scar, recurrence during follow up, and adverse events (AEs).

**Results:**

Of the 14 patient cases reviewed, there was 100% technical success and residual malignancy (RM) found in 14%. Of the two patients with residual disease, one achieved R0 resection with EFTR whereas the other did not and subsequently underwent surgery with no histopathological evidence of malignancy in the resected tissue. There was one AE of rectal bleeding that did not require any surgical intervention or blood transfusions.

**Conclusions:**

EFTR could offer endoscopists a safe, efficacious, and minimally invasive mechanism for formal tumor (T) staging of malignancies found within polypectomy segments. Further studies with larger sample sizes are needed to assess outcomes in patients with residual neoplastic disease.

## Introduction

In 2024, colorectal cancer (CRC) is expected to remain as the United States’ second leading cause of cancer deaths 1 . Most colon cancers arise from colon polyps, sometimes found focally within endoscopically resected pieces of a specimen. Large polyps (> 20 mm) are often resected in multiple pieces, which is referred to as piecemeal polypectomy. For such patients, margins cannot be assessed, thus making further management decisions unclear because the stage is difficult to determine.

Often, these patients undergo surgical resection due to possible risk of nodal involvement from colon cancer progressing below the mucosa. Unfortunately, surgical intervention of non-malignant polyps leads to 14% of patients experiencing major postoperative events 2 , highlighting the need for less invasive approaches to CRC surveillance of suspicious lesions. In addition, the concurrent issue of our increasingly aging and morbid population underpins the need for minimally invasive treatment options for poor surgical candidates.

Decades of advancements in medical technology have led to numerous novel endoscopic resection techniques that expand endoscopist ability to intervene without subjecting patients to surgery. Endoscopic mucosal resection (EMR) and endoscopic submucosal dissection (ESD) are commonly used techniques, with ESD achieving higher rates of histologically complete (R0) resection when compared with EMR 3 4 5 6 . However, ESD is associated with longer procedure times and potentially higher adverse event (AE) rates due to greater technical complexity. Thus, widespread use of EMR often leads to incomplete removal of malignant colon polyps 4 5 6 7 . The newest addition to the endoscopist toolkit is endoscopic full-thickness resection (EFTR), which is an alternate approach to remove colon polyps when EMR may lead to incomplete resection and/or a case not amenable to ESD 7 8 9 .

In 2016, the US Multi-Society Task Force (USMSTF) provided updated guidelines for management of patients after CRC resection 10 . Given that most patients at that time underwent surgical resection, there is limited evidence about those who were endoscopically managed. A 2020 consensus update to the USMSTF recommendations suggests that surveillance colonoscopy following polypectomy of a high-risk lesion reduces incidence and mortality from CRC 11 . There are limited studies regarding endoscopic resection of malignant lesions and subsequent surveillance approaches, leaving physicians treating poor surgical candidates without clear guidelines for management.

We present a prospective cohort of patients who underwent piecemeal polypectomy or EMR of a malignant polyp with subsequent unclear margins or stage, who could not or did not wish to undergo surgery and opted for EFTR of the scar. The purpose of this study was to assess efficacy of EFTR in achieving R0 resection of incompletely resected, malignant colorectal lesions in nonsurgical candidates. We predicted that EFTR would provide a nonsurgical alternative to scar consolidation and that R0 resection rates would be comparable to EMR and ESD.

## Patients and methods

This was a single-center prospective cohort of individuals who underwent EFTR following removal of a malignant colon polyp at a major tertiary cancer center in the United States from January 2022 until September 2023. This study was approved by our Institutional Review Board (STUDY20201645 *)* and deidentified data were obtained prior to analysis, stored in an Excel file, and password protected. This study was internally funded, not by any EFTR device company and no authors had financial relationships with any EFTR device company.

**Fig. 1:**
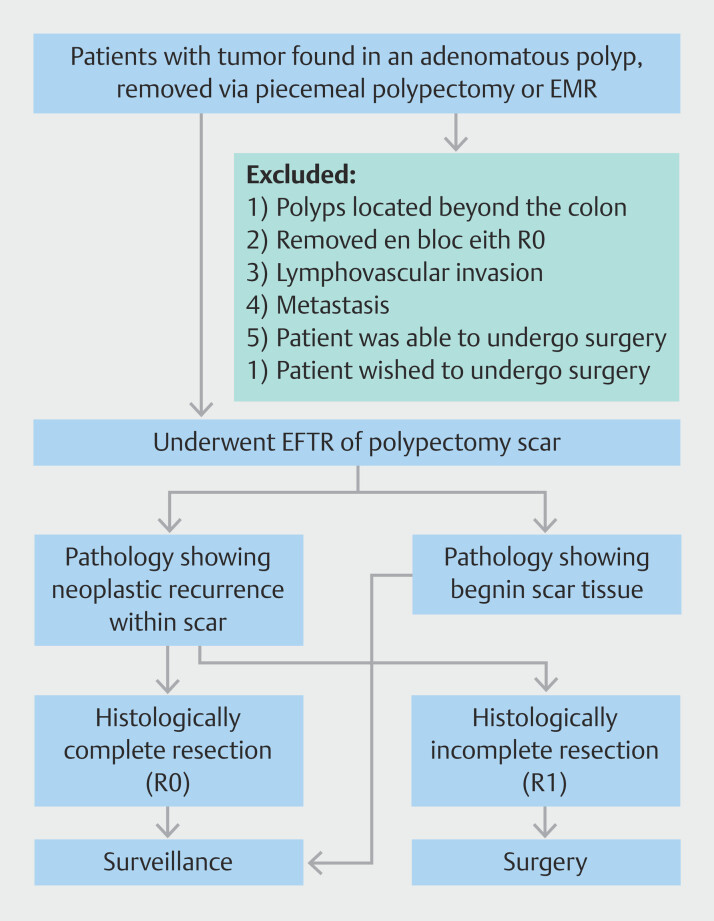
Patient sampling process and study design flowchart. EMR, endoscopic mucosal resection; EFTR, endoscopic full-thickness resection; R0, histologically complete; R1, histologically incomplete.

The inclusion criterion was patients 18 years of age or older with a history of piecemeal polypectomy or EMR of a malignant colorectal polyp who subsequently had EFTR of their polypectomy scar. All patients were not pregnant, and we only included subjects that did not wish to or could not undergo surgical resection. Patients were excluded if: 1) scars in question were in organs other than the colon; 2) initial en bloc resection was performed, obtaining an R0 result; 3) lymphovascular invasion (LVI) was present; 4) metastatic disease was present on imaging; 5) they were able to undergo surgery; or 6) they wished to undergo surgery ( Fig. 1 ). Variables collected include patient demographics, primary polyp features, EFTR procedure details, pathology of the EFTR margins, length of time between procedures, imaging results, and AEs.

The EFTR procedure involved navigating to the site of prior polypectomy via colonoscopy and marking the edges of the scar. Next, a grasper was extended from the full-thickness resection device to pull the lesion into the scope cap. Once the lesion and a portion of bilateral serosal tissue was fully within the cap, a metal clip was placed over the distal end of the scope, securing the serosal linings together. Lastly, an electrosurgical snare cut proximal to the clip and the lesion was removed within the scope cap. Margin biopsies were obtained with cold forceps. Technical success was defined as complete gross resection of the polypectomy scar with one clip placement.

Primary outcomes of this study were R0 resection and technical success of EFTR. Secondary outcomes include prevalence of resection margin (RM) after piecemeal polypectomy, recurrence during follow-up, and AEs.

Because this was a prospective series without a control group, descriptive statistics were utilized for all demographic and resection data using standard methods to determine means.

## Results

From January 2022 until September 2023, 14 cases were performed at our institution, nine (64%) were male, and 13 (93%) were white. The most common polyp location was in the rectum (64%) and 79% were adenocarcinoma within a tubular adenoma ( Table 1 ). A comprehensive summary of each patient in the sample can be seen in Table 2 . Mean polyp size was 23.0 mm and mean scar size was 10.1 mm.

**Table:** **Table 1** Summary of patient sample demographics and primary polyp features (N = 14).

Descriptor	N (%) Mean (SD)
**Total**	14 (100)
**Age**	64.4 (10.3)
**Sex**	
Male	9 (64)
Female	5 (36)
**Ethnicity**	
White	13 (93)
Hispanic/Latino	1 (7)
**Primary polyp location**	
Rectum	9 (64)
Sigmoid	3 (21)
Rectosigmoid	1 (7)
Transverse	1 (7)
**Primary polyp histology**	
Carcinoma within polyp	11 (79)
NET	3 (21)
**Histologic grade**	
Moderately differentiated	7 (50)
Well-differentiated	3 (21)
Poorly differentiated	3 (21)
Data unavailable	1 (7)
**Polyp size (mm)**	23 (11.9)
Values are presented as a number (percentage) or a mean (standard deviation). NET, neuroendocrine tumor; SD, standard deviation.	

**Table:** **Table 2** Descriptive statistics of the primary polyp pathology, EFTR procedure, and malignant residual disease.

Patient number	Polyp location in colon	Polyp size (mm)	Polyp histology	Polyp grade (differentiated)	Scar size (mm)	EFTR procedure duration (min)	RM tumor stage	R0 resection	EFTR success	Adverse event
1	Transverse	35	Tubular	Poorly	25	72	Tis	Yes	Yes	No
2	Rectum	N/A	N/A	Moderately	10	49	T2	No	Yes	No
3	Rectum	20	Neuroendocrine	Well	20	48	-	Yes	Yes	Yes
4	Sigmoid	20	Tubular	Moderately	3	30	-	Yes	Yes	No
5	Rectum	10	Neuroendocrine	Moderately	6	35	-	Yes	Yes	No
6	Rectum	12	Unknown	Moderately	6	40	-	Yes	Yes	No
7	Rectum	30	Tubulovillous	Poorly	8	39	-	Yes	Yes	No
8	Rectum	30	Tubulovillous	Moderately	8	69	-	Yes	Yes	No
9	Rectum	15	Tubular	Moderately	6	36	-	Yes	Yes	No
10	Sigmoid	40	Tubular	Poorly	10	28	-	Yes	Yes	No
11	Rectosigmoid	20	Unknown	Moderately	10	43	-	Yes	Yes	No
12	Rectum	N/A	Tubulovillous	N/A	6	17	-	Yes	Yes	No
13	Sigmoid	40	Tubulovillous	Moderately	8	39	-	Yes	Yes	No
14	Rectum	4	Neuroendocrine	Well	5	35	-	Yes	Yes	No
Depth of invasion of the initial malignancy could not be determined due to resection by piecemeal polypectomy. Tumor stage was only reported in the cases where RM was found within the scar by EFTR. EFTR, endoscopic full-thickness resection; RM, residual malignancy; R0, histologically complete.										

**Fig. 2:**
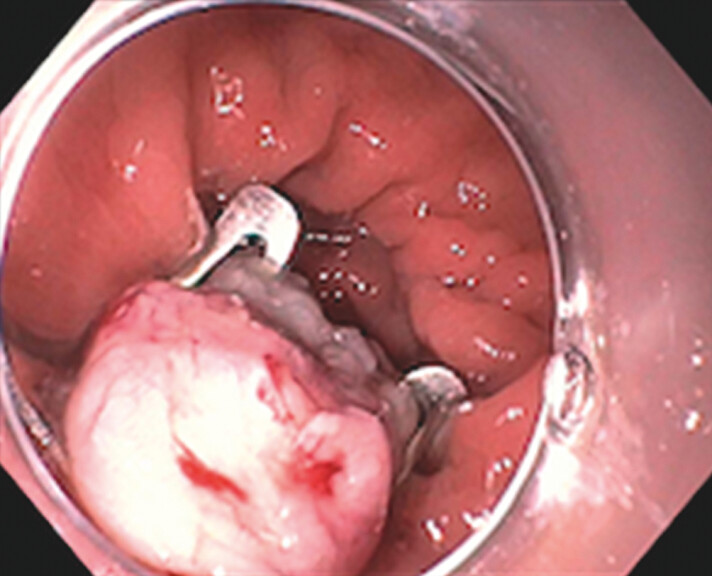
Scar consolidation site after EFTR with the scope clip securing the mucosa. EFTR, endoscopic full-thickness resection.

Of the 14 patient cases reviewed, there was 100% technical success with mean procedure time being 41.4 minutes for EFTR ( Table 3 ). Fig. 2 shows an example image from the EFTR of one of our study patients. There were two cases with RM found within the scar (RM rate, 14%). They were staged at Tis and T2. R0 resection was achieved in one of these patients and the second patient then was agreeable to colectomy. In addition, there was one AE reported (7%) in the sample. To better understand the ideal patient population for EFTR, we will detail these cases below.

**Table:** **Table 3** Summary of EFTR outcomes (N = 14).

Measures	Outcomes
Technical success, (%)	14, (100)
Mean procedure time, (SD)	41.4 min, (14.8)
Mean scar size, (SD)	10.1 mm, (6.0)
Median time piecemeal polypectomy and EFTR, (IQR)	14.8 weeks, (12.4–27.1)
RM, (%)	2, (14)
R0 resection, (%)	13, (93)
Adverse events, (%)	1, (7)
Values are presented as a number (percentage), a mean (standard deviation), or a median (interquartile range). EFTR, endoscopic full-thickness resection; IQR, interquartile range; RM, residual malignancy; R0, histologically complete; SD, standard deviation.	

### Case series highlights

Patient one, whose scar had Tis RM and was completely resected by EFTR, was a 71-year-old White man. His primary polyp was 35 mm with a small focus of poorly differentiated, intramucosal adenocarcinoma located in the transverse colon. This polyp was removed via piecemeal EMR with hot avulsion and was morphologically described as a Paris IIa+ IIc lesion. The EFTR procedure took 72 minutes and the size of the scar removed was 25 mm. Given the intramucosal location and minute size (0.5 mm) of the tumor, the final stage was determined to be carcinoma in situ. The time elapsed between piecemeal polypectomy and EFTR was 4.4 months. Baseline imaging showed pelvic lymphadenopathy, for which a retroperitoneal core needle biopsy was performed and showed benign lymph node tissue. On follow-up imaging 2 years later, lymphadenopathy had decreased in size and lacked concerning features. Workup for this lymphadenopathy revealed that the patient has a genetic variant (CTLA4 pathogenic mutation) of autoimmune lymphoproliferative syndrome.

The patient whose scar had RM and did not achieve R0 resection with EFTR was a 66-year-old Hispanic physician. His primary polyp was in the rectum and the adenocarcinoma found within it was moderately differentiated. The size and histology were not available on outside records. Time between piecemeal polypectomy and EFTR was 1.2 months. His procedure took 49 minutes and the size of the scar removed was 10 mm. Cold forceps were used for margin biopsies. The RM showed residual carcinoma in the muscularis propria (T2), leading the patient to undergo a low anterior resection surgery. Surgical pathology showed no residual carcinoma in the scar or rectum.

Regarding AEs, there was one in a 69-year-old White woman who underwent EFTR of a 20-mm well-differentiated rectal neuroendocrine tumor scar. Time elapsed between baseline piecemeal polypectomy and EFTR was 13.8 months, with an EMR procedure performed in between. She presented to the hospital for rectal bleeding 3 weeks after her EFTR procedure. Flexible sigmoidoscopy was performed to assess the procedure site, revealing a clean-based ulcer without active bleeding, and no over-the-scope clip was seen. No additional intervention was required. The patient was hemodynamically stable throughout the hospitalization and required no transfusions.

## Discussion

### Lifting the burden of surgery

Performing elective colectomies for nonmalignant colorectal polyps is a source of significant patient morbidity and mortality, while also contributing excessive costs to the healthcare system 2 . The annual cost of elective colectomies is over $1.7 billion, with 11.3% being due to postoperative AEs 12 . In this study, 93% of the patient sample achieved a clear margin from their malignant polyp scar while avoiding surgery and with limited AEs. It is important to note that the patient who underwent surgery had no residual carcinoma found within the tissue despite being staged at T2. When a patient with stage T2 CRC also has lymph node metastasis (LNM), they are significantly more likely to experience recurrence than those without LNM, specifically in the form of liver metastasis 13 . Independent risk factors for nodal involvement in stage T2 CRC include poorly differentiated grade and presence of LVI 13 14 15 . Given risk of nodal involvement for T2 colorectal cancers, surgery is the most appropriate intervention; however, in our case, it appears that EFTR would have led to a curative result. A new scoring system to assess risk of local LNM from early-stage rectal cancer has been proposed to help clarify decision making for such patients 14 .

### Current endoscopic resection techniques

The USMSTF guidelines for endoscopic CRC surveillance assume complete resection of an initial lesion; however, studies show that 19% of CRC is attributable to incomplete resection on baseline colonoscopy using polypectomy or EMR 11 12 . These approaches are significant risk factors for local recurrence 13 14 15 16 , highlighting the need for better resection and surveillance strategies to reduce incidence of CRC in post-piecemeal polypectomy and EMR patients.

It is important to compare existing techniques to EFTR to determine the most efficacious, safe, and cost-effective approach to achieving R0 resection. ESD is a common endoscopic approach used for en bloc resection, with documented high R0 resection (84.2%) and low recurrence (5.8%) 17 . Use of complementary techniques, such as ESD with margin ablation, have recently been found to boost efficacy further 18 . The primary limitation of this approach is its relatively high rates of AEs and technical difficulty, resulting in limited availability of endoscopists trained in ESD 4 6 .

Compared with ESD, EFTR is less technically difficult and is similarly efficacious when used for en bloc resection; however, the device is limited by size and becomes less effective with lesions > 20 mm 8 19 . As a result, EFTR is noted to be most effective in removing non-lifting or fibrotic lesions, such as scars 3 . In our study, when clear margins were not obtained by EFTR, the surgical pathology report described no neoplastic disease in the specimen. Given that the margins were assessed through cold forcep biopsies after EFTR, this may indicate that a combination approach with the EFTR and margin ablation could improve outcomes, as seen with ESD.

### CRC surveillance recommendations

In 2020, the European Society of Gastrointestinal Endoscopy (ESGE) published a recommendation for follow-up surveillance colonoscopy within 6 months of piecemeal polypectomy of a polyp > 20 mm 3 . This recommendation addresses the concern for histologically incomplete (R1) resection of these high-risk lesions but fails to specify how to manage the polypectomy scar, if at all. Endoscopic ultrasound is used for staging colorectal tumors; however, when used for scar surveillance, it has not been shown to be effective 20 . Considering the ESGE guideline and our 100% technical success with EFTR in post-piecemeal polypectomy or EMR scar consolidation, this study exemplifies the potential benefit of using EFTR for short-term surveillance.

### Benefits, limitations, and future directions

Using EFTR for scar consolidation of colorectal lesions with unclear resection margins offers patients formal tumor (T) staging without surgical intervention. This lessens the physical, emotional, and financial toll of surgery on the patient, while also lifting the burden of elective colectomies for nonmalignant lesions from the healthcare system.

The one AE that occurred highlights a potential risk of EFTR. The patient underwent piecemeal polypectomy about 14 months prior and an EMR 7 months prior to EFTR, likely weakening the mucosal integrity and ultimately contributing to the clip falling out. This did not cause a perforation or require any significant intervention, but rather, exemplifies risk of over-manipulation of tissue prior to EFTR. Emergent colectomies have significantly higher morbidity and mortality rates when compared with elective colectomies (48.4% vs 25.6%) 21 , highlighting the importance of understanding the risk profile of EFTR. More studies are needed to understand the safe time frame and frequency of endoscopic intervention prior to EFTR.

Primary limitations of this study are small sample size and reduced specificity of nodal (N) staging, due to inability to biopsy regional lymph nodes. Although using imaging to monitor for malignant lymph nodes is less reliable, we hope that through long-term surveillance and increasing sample sizes, clinicians will be assured that the benefit of this minimally invasive approach to formal T staging outweighs risks of reduced N staging specificity. Currently, our small sample size limits ability to draw conclusions about efficacy of EFTR in achieving R0 resection in cases where RM is found within the scar. In addition, our data collection regarding the primary polyp features was limited by variability in endoscopy and pathology reports obtained from external referral centers.

Future directions for this project will focus on increasing sample size and analyzing cases in which EFTR fails to achieve R0 resection. It would be useful to assess surgical pathology and nodal involvement of T2 lesions in those cases to better understand the true efficacy of EFTR. It would also be useful to assess efficacy of EFTR in other organs of significant fibrosis where there is concern for malignancy, such as previous surgical, ESD, or stenotic sites.

## Conclusions

Utility of EFTR in post-piecemeal polypectomy or EMR scar consolidation of neoplastic polyps shows promise by offering formal T staging of these high-risk lesions and limiting the burden of surgery with low AE rates. EFTR would be an ideal choice to confirm R0 resection of malignancy found within polyp fragments in a patient without signs of metastasis on imaging. A larger sample size is needed to determine efficacy of EFTR in achieving R0 resection in patients with RM.

## References

[1] American Cancer Society Cancer Facts & Figures 2024 https://www.cancer.org/research/cancer-facts-statistics/all-cancer-facts-figures/2024-cancer-facts-figures.html

[2] Peery AF Shaheen NJ Cools KS Morbidity and mortality after surgery for nonmalignant colorectal polyps Gastrointest Endosc 2018 87 243 250 e242 10.1016/j.gie.2017.03.1550 28408327 PMC5634910

[3] Atla PR Alao H Reicher S Endoscopic submucosal dissection versus endoscopic full-thickness resection for challenging colorectal lesions: Must we choose? Gastrointest Endosc 2023 98 998 999 10.1016/j.gie.2023.08.012 37977674

[4] Fujiya M Tanaka K Dokoshi T Efficacy and adverse events of EMR and endoscopic submucosal dissection for the treatment of colon neoplasms: a meta-analysis of studies comparing EMR and endoscopic submucosal dissection Gastrointest Endosc 2015 81 583 595 10.1016/j.gie.2014.07.034 25592748

[5] Ito S Hotta K Sekiguchi M Short-term outcomes of endoscopic resection for colorectal neuroendocrine tumors: A Japanese multicenter prospective C-NET STUDY Dig Endosc 2023 36 942 951 10.1111/den.14728 37986226

[6] Panzuto F Magi L Esposito G Comparison of endoscopic techniques in the management of type i gastric neuroendocrine neoplasia: A systematic review Gastroenterol Res Pract 2021 2021 6.679397E6 10.1155/2021/6679397 PMC8026302 33859684

[7] Dumoulin FL Hildenbrand R Endoscopic resection techniques for colorectal neoplasia: Current developments World J Gastroenterol 2019 25 300 307 10.3748/wjg.v25.i3.300 30686899 PMC6343101

[8] Albrecht H Raithel M Braun A Endoscopic full-thickness resection (EFTR) in the lower gastrointestinal tract Tech Coloproctol 2019 23 957 963 10.1007/s10151-019-02043-5 31368009

[9] Obri M Ichkhanian Y Brown P Full-thickness resection device for management of lesions involving the appendiceal orifice: Systematic review and meta-analysis Endosc Int Open 2023 11 E899 E907 10.1055/a-2131-4891 37810898 PMC10558260

[10] Kahi CJ Boland CR Dominitz JA Colonoscopy surveillance after colorectal cancer resection: recommendations of the US Multi-Society Task Force on Colorectal Cancer Gastrointest Endosc 2016 83 489 498 e410 10.1053/j.gastro.2016.01.001 26802191

[11] Gupta S Lieberman D Anderson JC Recommendations for follow-up after colonoscopy and polypectomy: A consensus update by the US Multi-Society Task Force on Colorectal Cancer Am J Gastroenterol 2020 115 415 434 10.1016/j.gie.2020.01.014 32039982 PMC7393611

[12] Robertson DJ Lieberman DA Winawer SJ Colorectal cancers soon after colonoscopy: a pooled multicohort analysis Gut 2014 63 949 956 10.1136/gutjnl-2012-303796 23793224 PMC4383397

[13] Belderbos TD Leenders M Moons LM Local recurrence after endoscopic mucosal resection of nonpedunculated colorectal lesions: systematic review and meta-analysis Endoscopy 2014 46 388 402 10.1055/s-0034-1364970 24671869

[14] Ohata K Kobayashi N Sakai E Long-term outcomes after endoscopic submucosal dissection for large colorectal epithelial neoplasms: A prospective, multicenter, cohort trial from Japan Gastroenterology 2022 163 1423 1434 e1422 35810779 10.1053/j.gastro.2022.07.002

[15] Hotta K Saito Y Matsuda T Local recurrence and surveillance after endoscopic resection of large colorectal tumors Dig Endosc 2010 22 S63 S68 10.1111/j.1443-1661.2010.00965.x 20590775

[16] Komeda Y Watanabe T Sakurai T Risk factors for local recurrence and appropriate surveillance interval after endoscopic resection World J Gastroenterol 2019 25 1502 1512 10.3748/wjg.v25.i12.1502 30948913 PMC6441916

[17] Draganov PV Aihara H Karasik MS Endoscopic submucosal dissection in north america: A large prospective multicenter study Gastroenterology 2021 160 2317 2327 e2312 10.1053/j.gastro.2021.02.036 33610532 PMC8783061

[18] Rotermund C Djinbachian R Taghiakbari M Recurrence rates after endoscopic resection of large colorectal polyps: A systematic review and meta-analysis World J Gastroenterol 2022 28 4007 4018 10.3748/wjg.v28.i29.4007 36157546 PMC9367239

[19] Lima Capela T Ferreira AI Macedo Silva V Recurrence rate after piecemeal endoscopic mucosal resection of <20 mm non-pedunculated colorectal lesions: should we worry about the risk? Scand J Gastroenterol 2023 59 361 368 37970898 10.1080/00365521.2023.2278425

[20] Stier MW Chapman CG Shamah S Endoscopic resection is more effective than biopsy or EUS to detect residual rectal neuroendocrine tumor Endosc Int Open 2021 9 E4 E8 10.1055/a-1300-1017 33403229 PMC7775810

[21] El Edelbi M Abdallah I Jaafar RF Comparing emergent and elective colectomy outcomes in elderly patients: A NSQIP study Int J Surg Oncol 2021 2021 9.990434E6 10.1155/2021/9990434 PMC8668335 34912578

